# Supplementary material for: Genetic Diversity and Population Structure of Common Bean (Phaseolus vulgaris L.) Landraces in the Lazio Region of Italy
Source: Plants (Basel). 2023 Feb 7;12(4):744. doi: 10.3390/plants12040744 (PMC9968208; doi:10.3390/plants12040744)
Supplement: Supplementary file 1 [file plants-12-00744-s001.zip › plants-2143655-supplementary/Supplementary materials.pdf]

Additional Figures

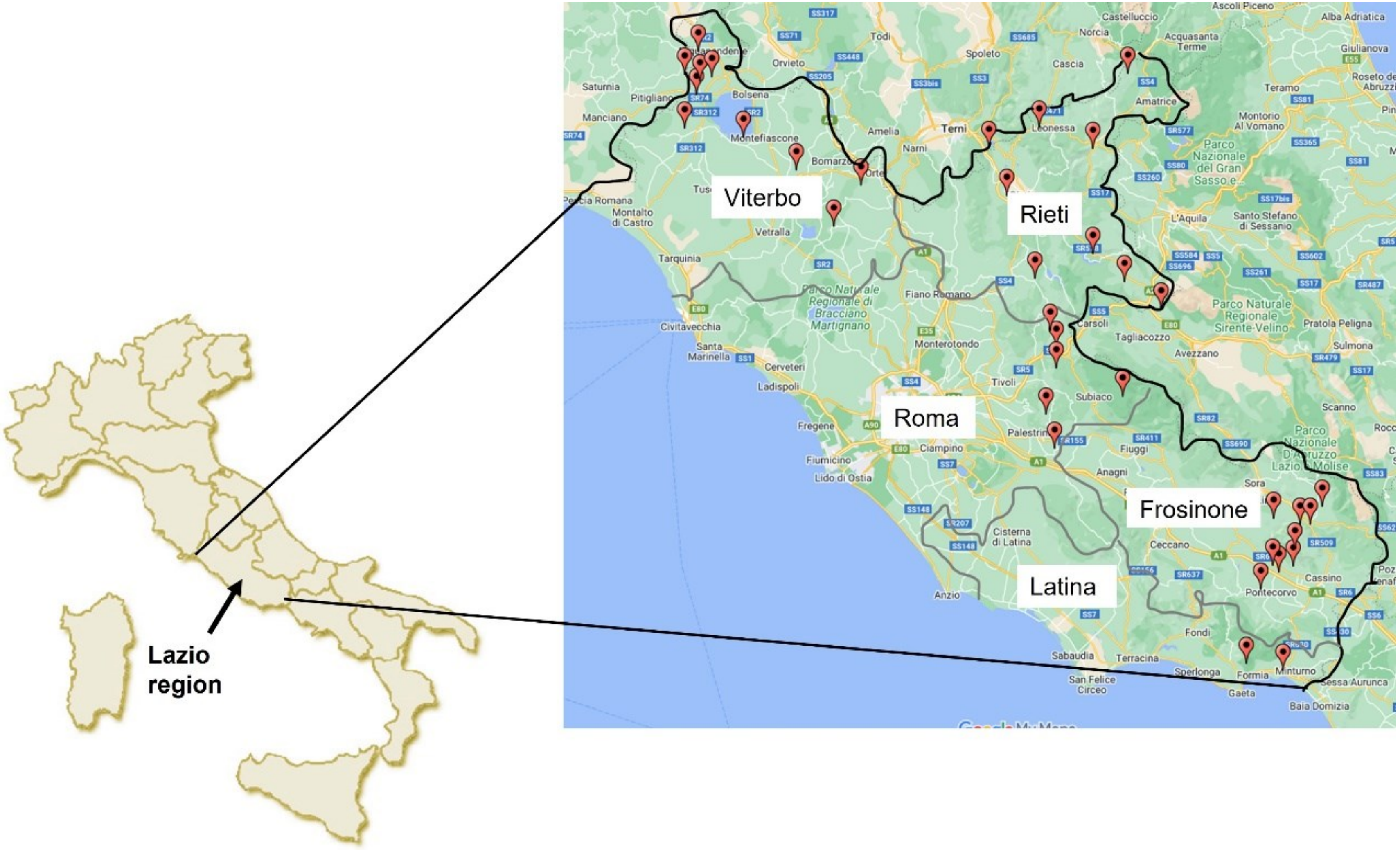

Figure S1. Locations of origin of the 114 accessions in the five provinces of the Lazio Region (adapted from Google Maps).

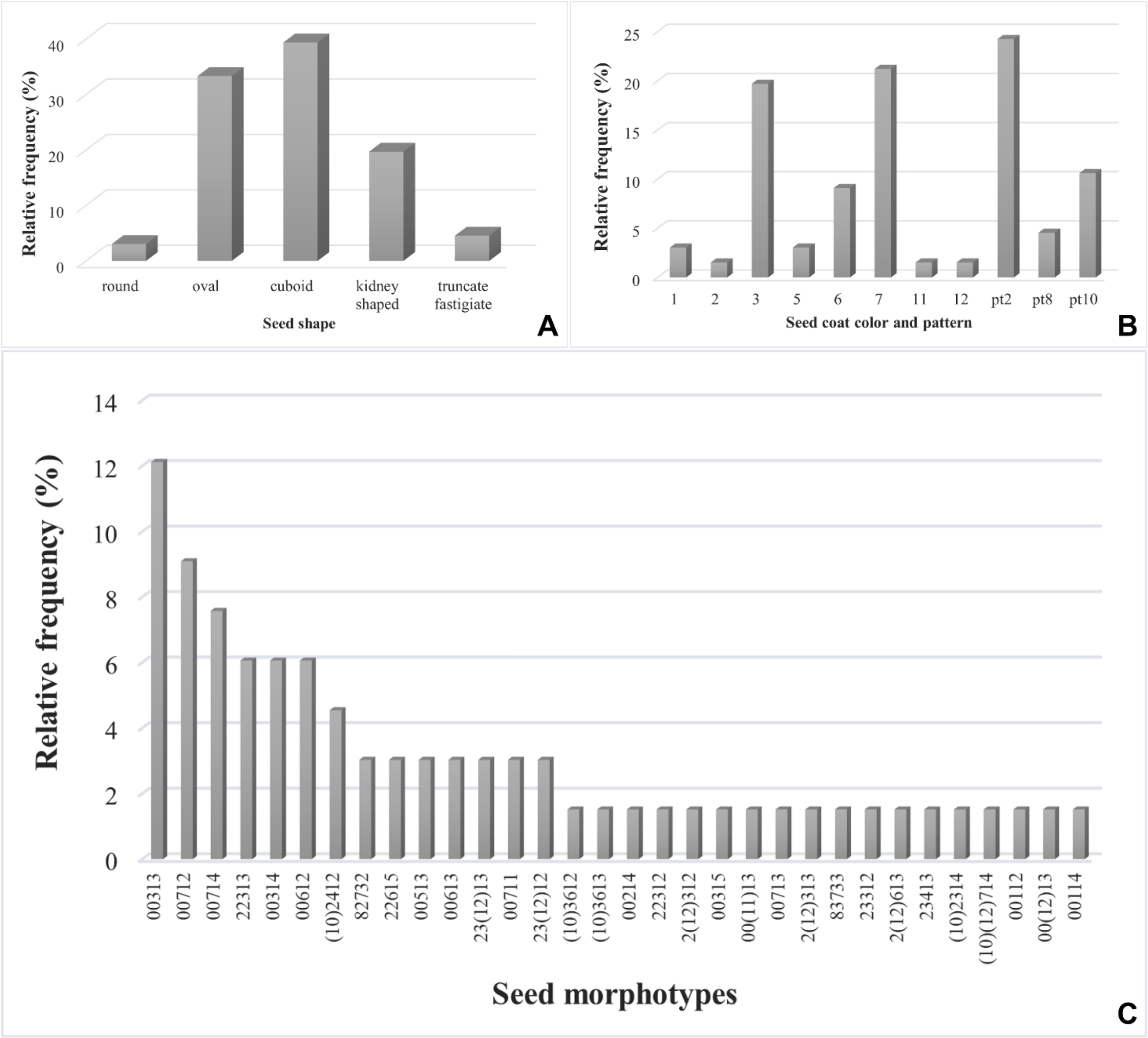

Figure S2. Frequency distribution of seed shape (A), seed coat color and pattern (B) and "seed morphotypes" (C) within the 66 common bean landraces collected from different areas of Lazio region. In the panel B in addition to the colors detected for the plain coat seeds (indicated by numbers according to the IPBGR descriptors), the different types of seed coat patterns (pt) are also reported. 1= black; 2= brown, pale to dark; 3= maroon; 5= yellow to greenish yellow; 6= pale-cream to buff (6); 7= pure white; 11= green to olive; 12= red; pt2= striped; pt8= bicolor; pt10= pattern around hilum.

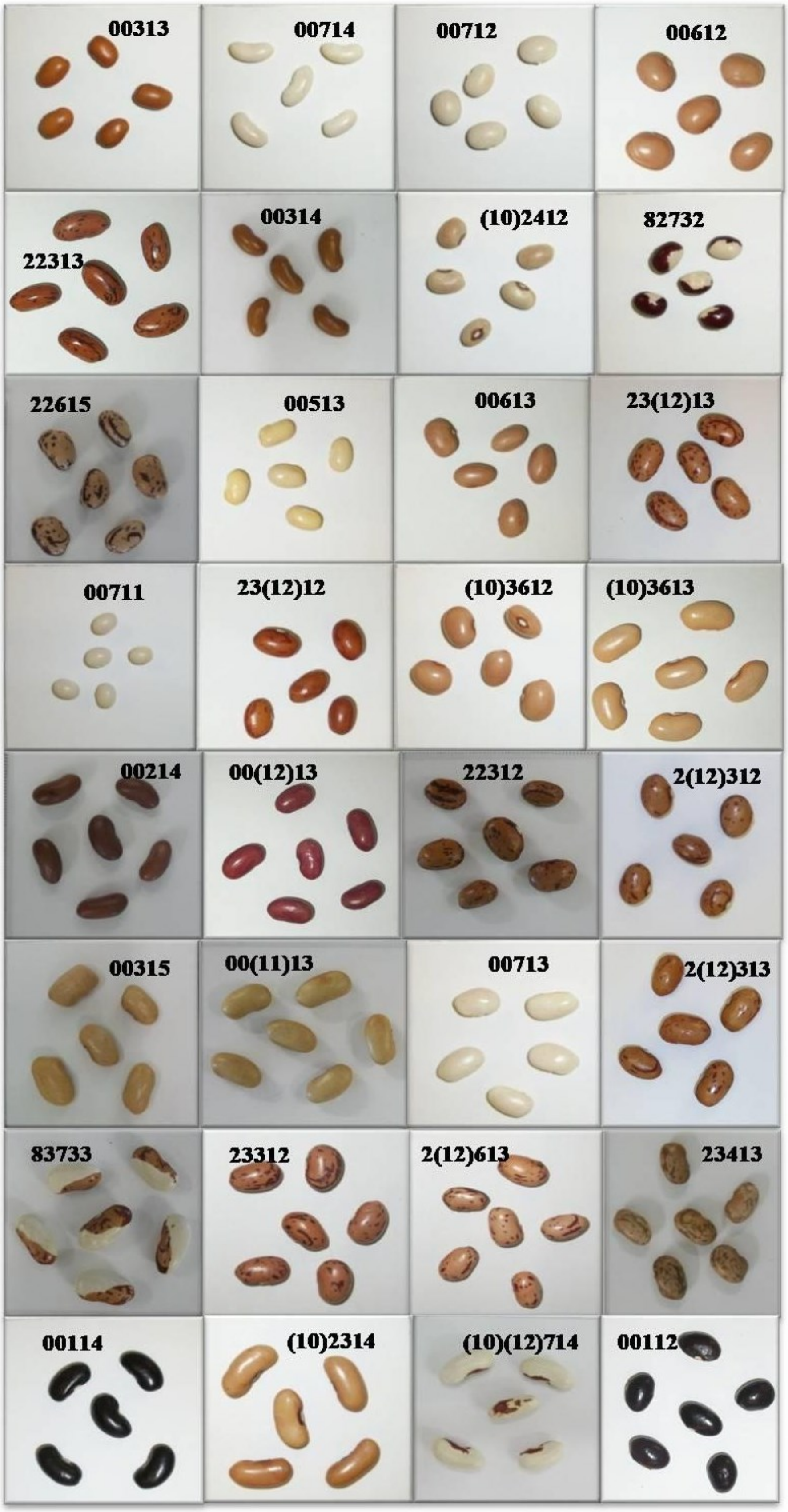

Figure S3. The 32 "seed morphotypes" identified for the 66 common bean landraces collected from different areas of the Lazio Region.

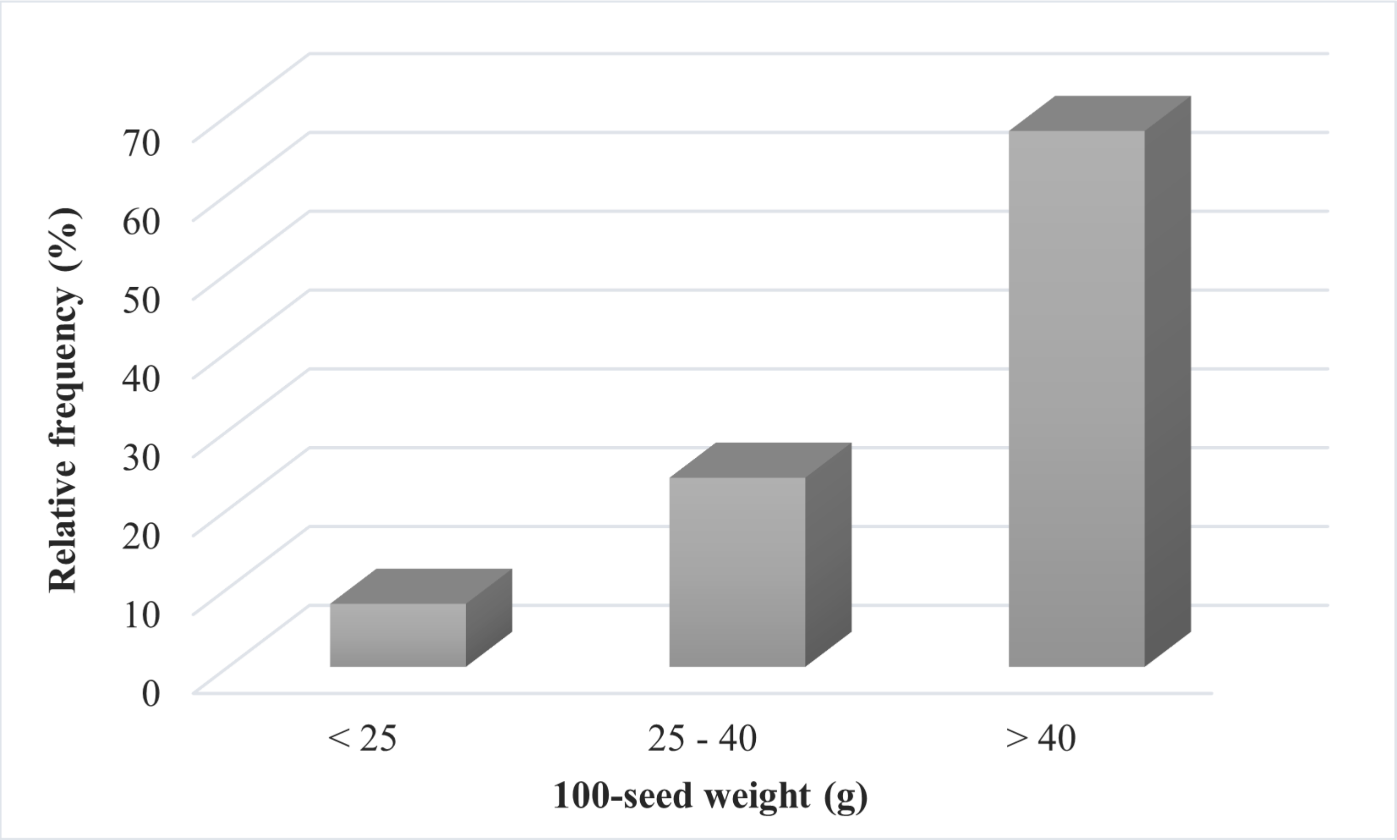

Figure S4. Frequency distribution of 100-seed weight within the 66 common bean landraces collected from different area of the Lazio Region.

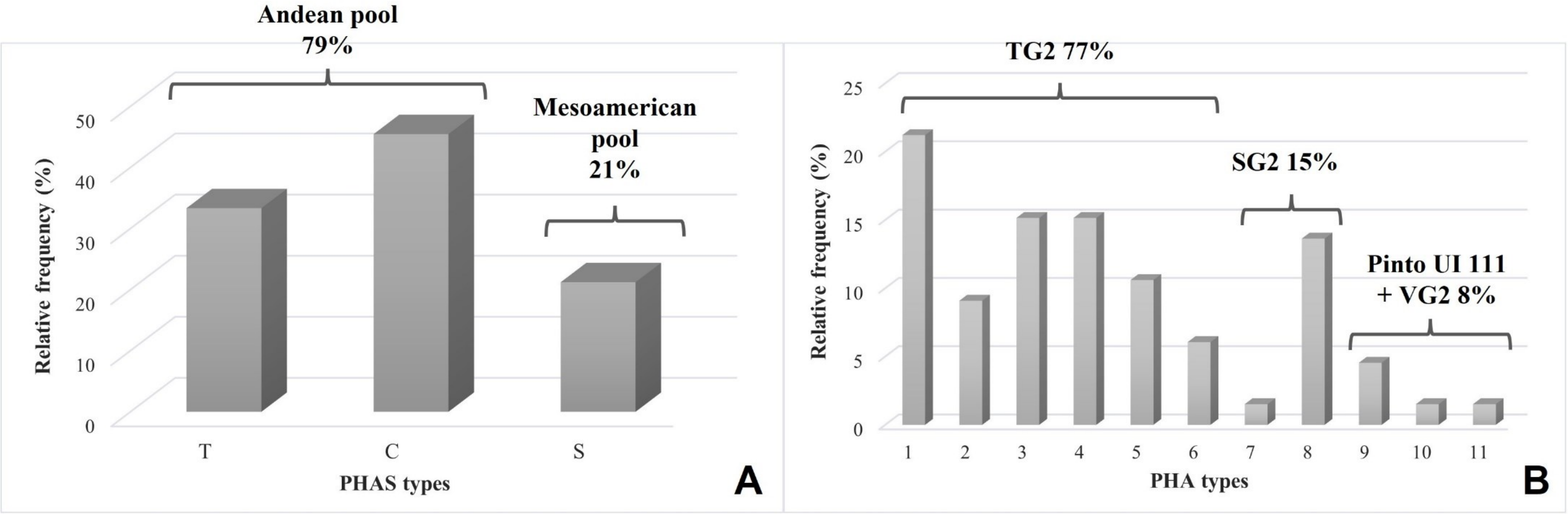

Figure S5. Frequency distribution of PHAS (A) and PHA (B) protein patterns within the 66 common bean landraces from the Lazio region. In the panel A is indicated the relative frequency of the C and T PHAS types associated to the Andean pool and that of the S PHAS type typical of the Mesoamerican pool. In the panel B are reported the relative frequencies of the four major variant groups TG2, SG2, Pinto UI 111 and VG2 [67], by which the 11 identified PHA patterns can be divided.

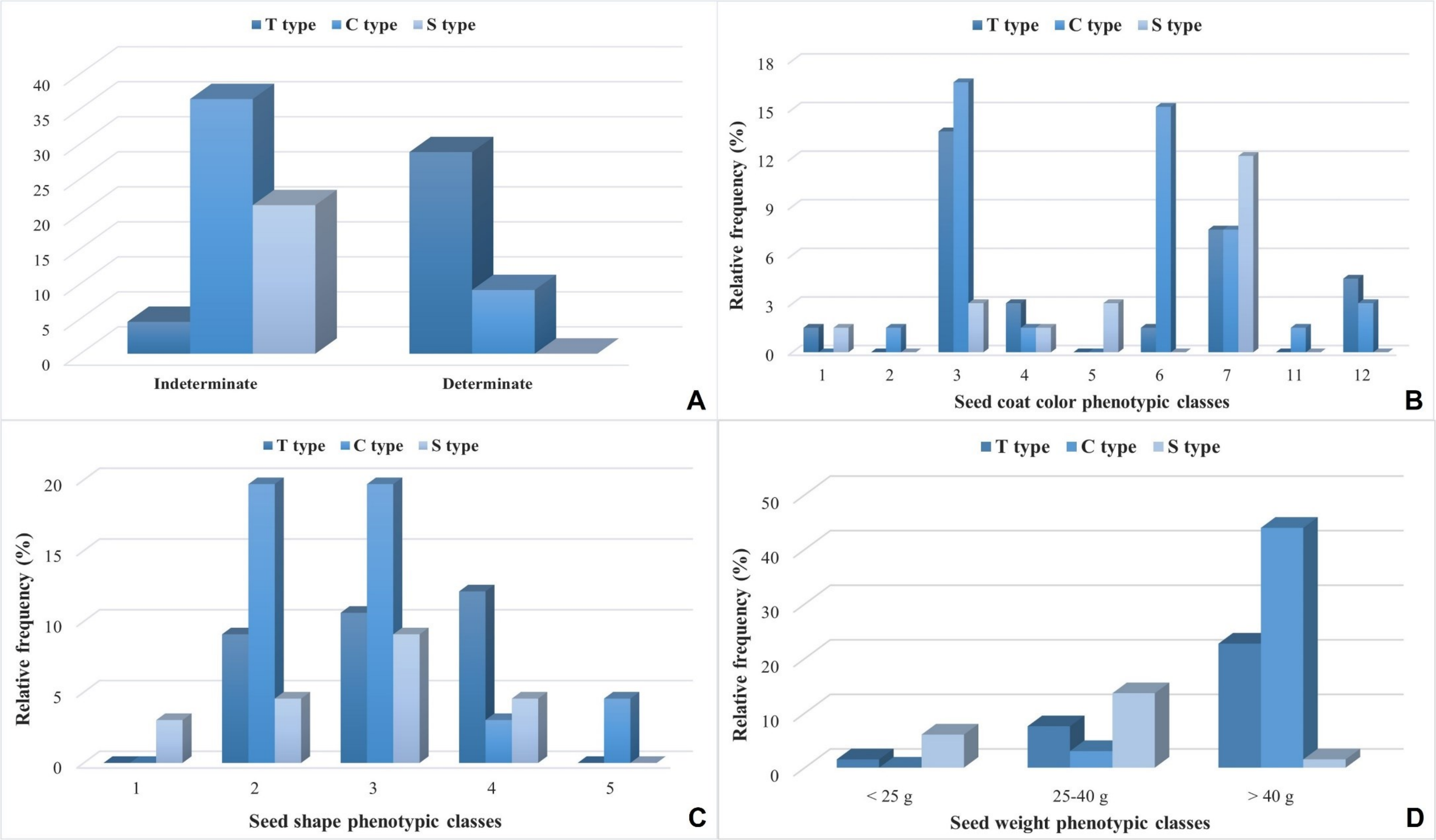

Figure S6. Frequency distribution of morphological traits in relationship with PHAS types in the landrace collection from Lazio region. A) Plant type: determinate and indeterminate growth habit. B) Seed coat color: 1= black; 2= brown, pale to dark; 3= maroon; 4= grey, brown to greenish; 5= yellow to greenish yellow; 6= pale-cream to buff (6); 7= pure white; 11= green to olive; 12= red. C) Seed shape: 1= round; 2= oval; 3= cuboid; 4= kidney shaped; 5= truncate fastigiated. D) Seed size: small (100 seed weight < 25 g), medium (25-40 g) and large (> 40 g).

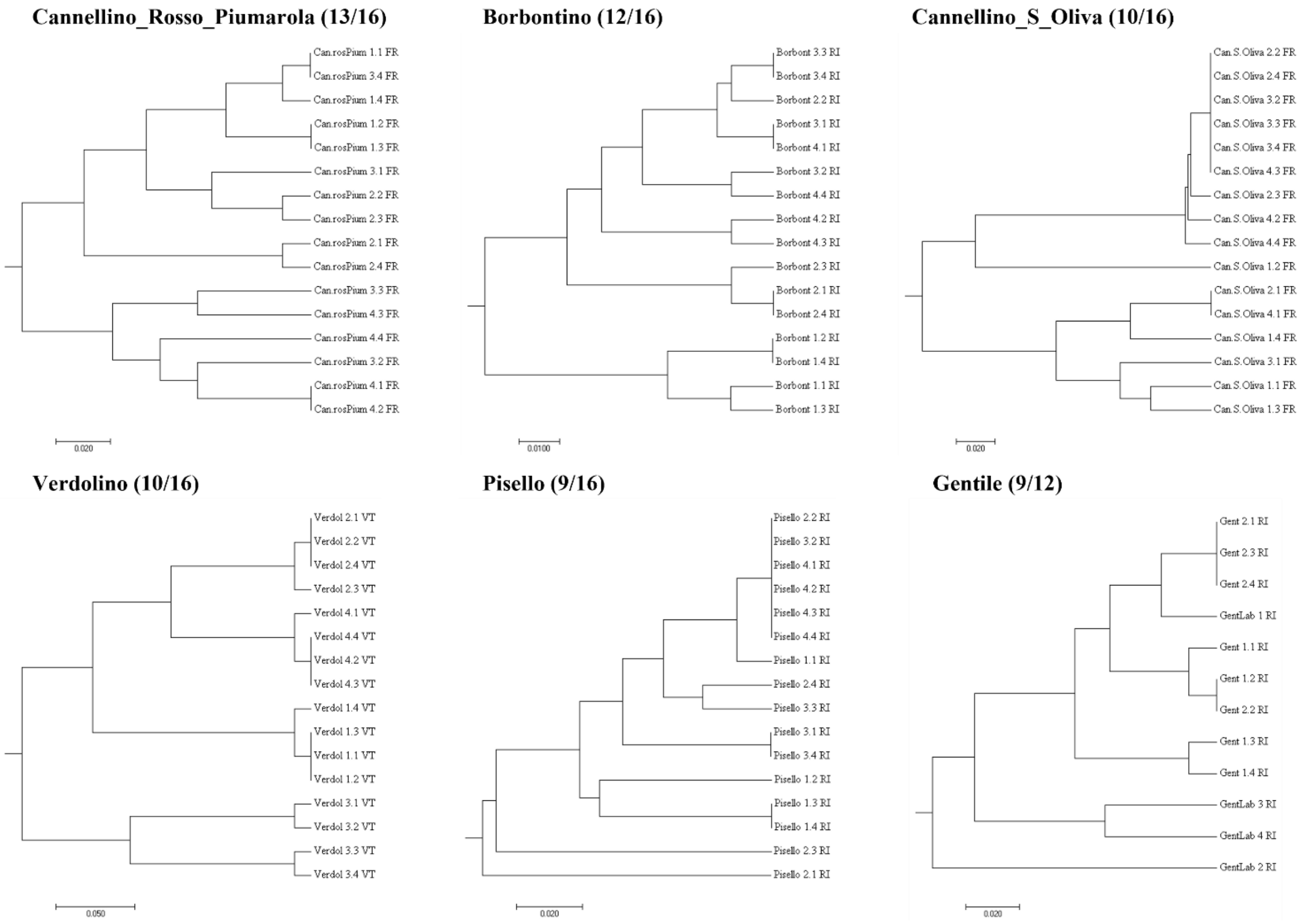

Figure S7. UPGMA trees based on Nei's coefficient [59] among the different plants analysed for six landraces showing the unique genotypes identified. For each landrace, the different genotypes on the total plants analysed are indicated in brackets.

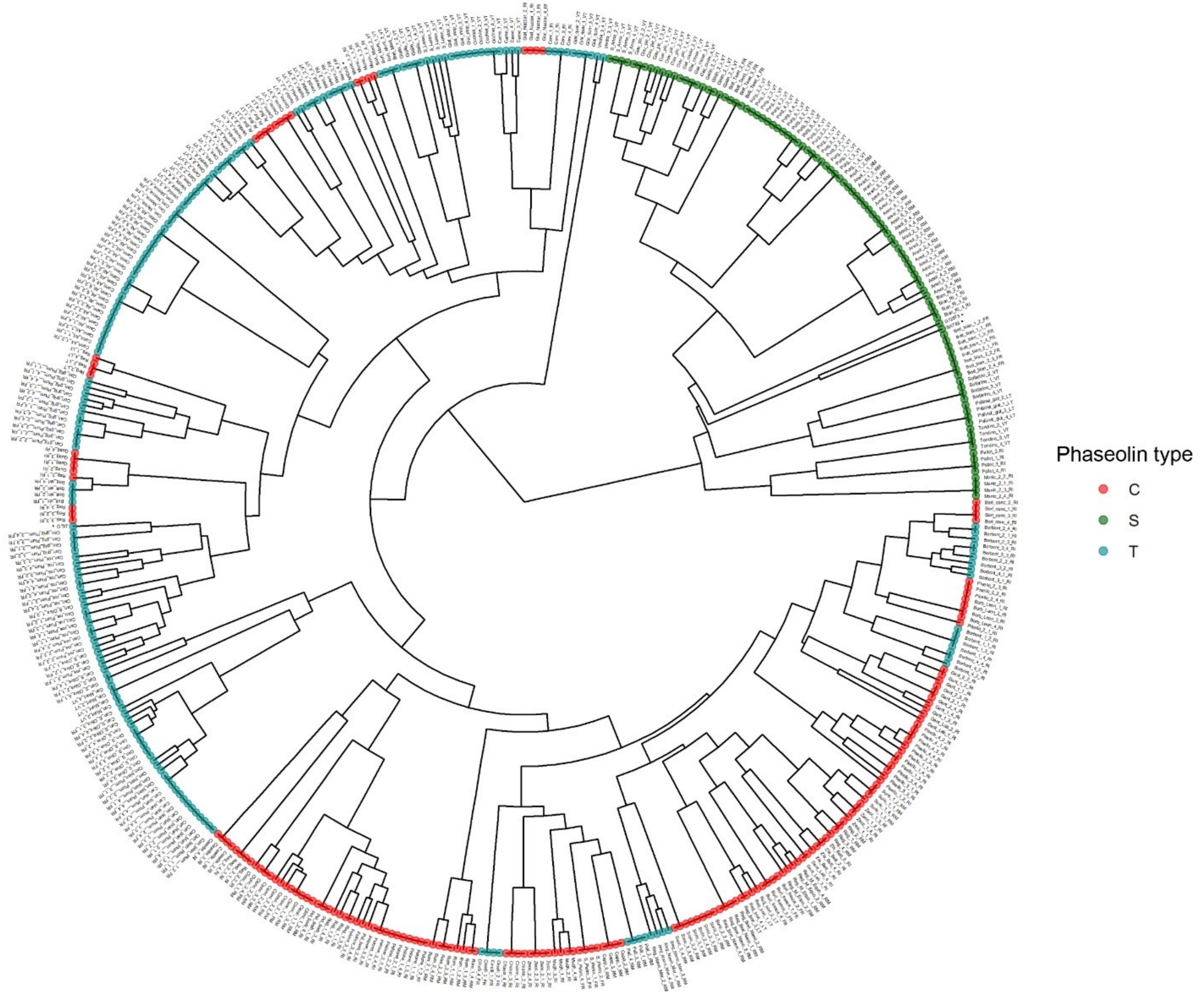

Figure S8. UPGMA tree based on Nei's coefficient [59] among the 456 genotypes (four plants for each of the 114 accessions belonging to 66 different landraces from the Lazio Region). In the phylogenetic tree, the red, light blue, and green circles indicate the genotypes with C, T and S PHAS types, respectively. The genotypes BAT and G12873 of Mesoamerica origin and MIDAS and JALO of Andean origin were included as references.

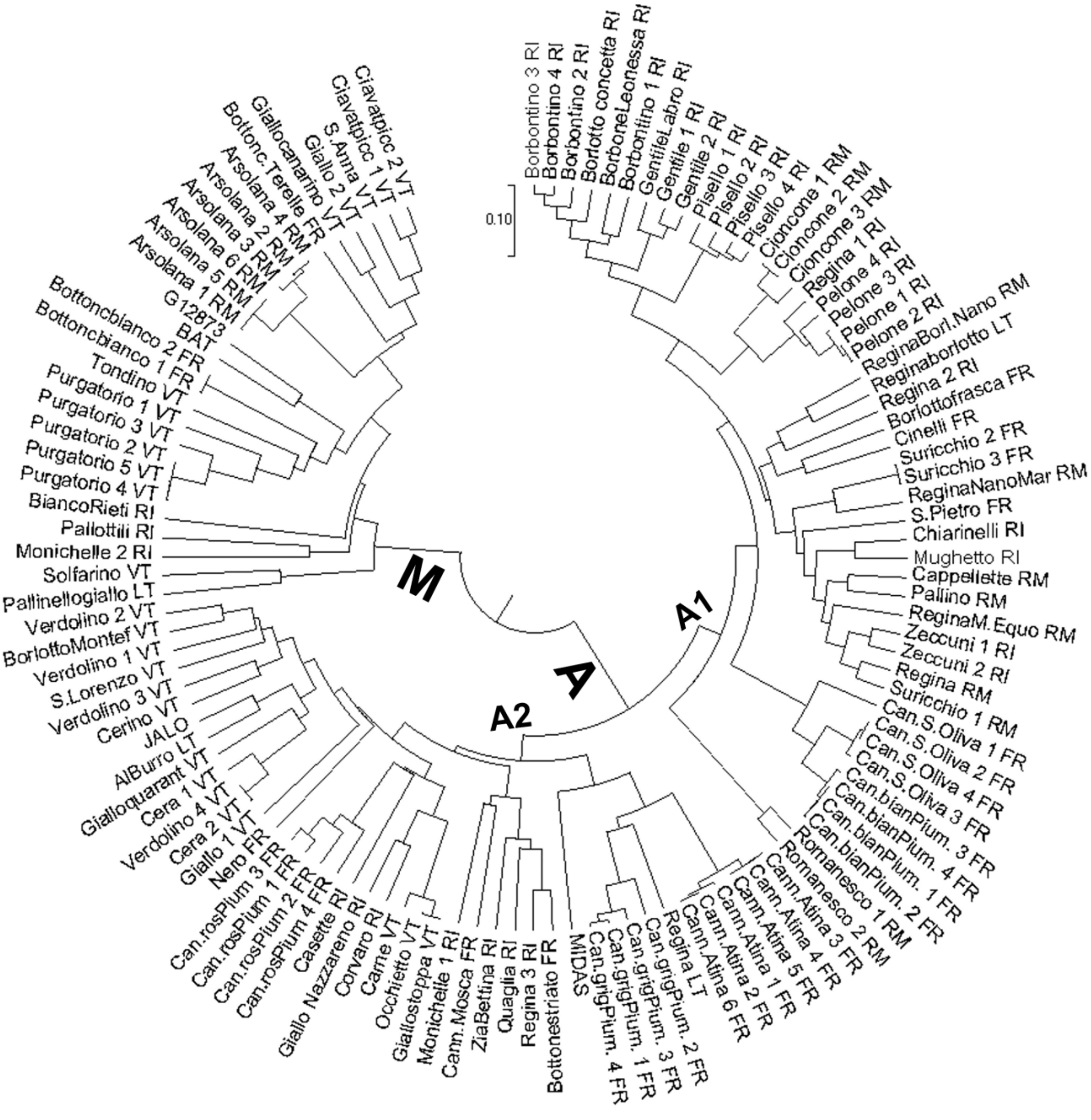

Figure S9. UPGMA tree based on Nei's coefficient [59] among the 114 accessions belonging to the 66 *P. vulgaris* landraces from Lazio Region. The genotypes BAT and G12873 of Mesoamerica origin and MIDAS and JALO of Andean origin were included as references.

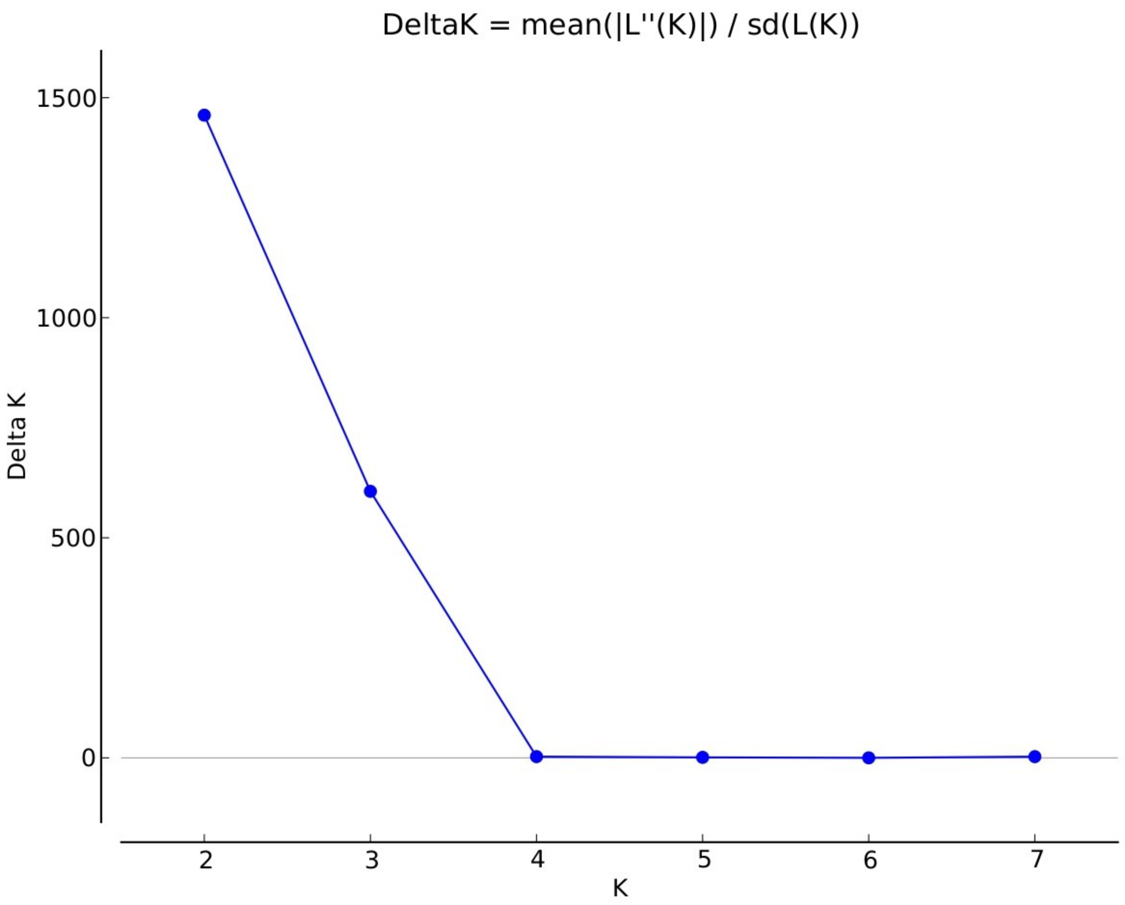

Figure S10. Estimation of the optimum number of clusters for the *P. vulgaris* genotypes according to the Evanno's method. The graph displays the DeltaK [mean(|L''(K)|) / sd(L(K))] for each K value.

Additional Tables

Table S2. Characteristics of the 12 microsatellite (SSR) markers used in this study.

SSR Code	Genebank entry	Description	LG	Forward primer	Reverse primer	Motif	Annealing (°C)	Dye color	References
PV-ag003	X04001	Glutamine synthetase	B01	TCACGTACGAGTTGAATCTCAGGAT	GGTGTCGGAGAGGTTAAGGTTG	$(AG)_8$	49	FAM	Yu et al. [50]
BMd-20	X74919	Endochitinase	B05	CCGTTGCCTGTATTTCCCCAT	CTGGTGAAGTCATCTGGAGTGGTC	$(AT)_5$	49	JOE	Yu et al. [50]
PV-at007	X80051	NADP-dependent malic enzyme	B09	AGTTAAATTATACGAGGTTAGCCTAAATC	CATTCCCTTCACACATTCACCG	$(AT)_{12}$	49	TAMRA	Yu et al. [50]
PV-at003	X60000	Small subunit of ribulose bisphosphate carboxylase/oxygenase	B04	ACCTAGAGCCTAATCCTTCTGCGT	GAATGTGAATATCAGAAAGCAAATGG	$(AT)_4(T)_2$	49	JOE	Yu et al. [50]
PV-ag001	M75856	Pathogenesis-related protein 3	B11	CAATCCTCTCTCTCTCATTTCCAATC	GACCTTGAAGTCGGTGTCGTTT	$(GA)_{11}$	49	FAM	Yu et al. [50]
PV-at004	X61293	Plastid-locatedglutamine synthetase (promotor region)	B06	AATCTGCCGAGAGTGGTCCTGC	GATTGAAATATCAAAGAGAATTGTTACC	$(AT)_{18}$	48	TAMRA	Yu et al. [50]
SHP1B		Genomic clone forthe SHATTERPROOF gene	B06	GGAAATTGAGCTGCAAAACC	CACAGTGTTCCCTGCATCAT		52	FAM	Nanni et al. [51]
BM160	AF483876	*P. vulgaris* genomic clone D137	B07	CGTGCTTGGCGAATAGCTTTG	CGCGGTTCTGATCGTGACTTC	$GA)_{15}(GAA)$	52	FAM	Gaitan-Solis et al. [52]
BM210	AF483902	*P. vulgaris* genomic clone D922	B07	CCCTCATCCTCCATTCTTATCG	ACCACTGCAATCCTCATCTTTG	$(CT)_{15}$	52	TAMRA	Gaitan-Solis et al. [52]
BM172	AF483884	*P. vulgaris* genomic clone D270	B03	CTGTACCTCAAACAGGGCACT	GCAATACCGCCATGAGAGAT	$(GA)_{23}$	52	JOE	Gaitan-Solis et al. [52]
BMd-12	AZ044945	*P. vulgaris* genomic clone Bng225/R	B06	CATCAACAAGGACAGCCTCA	GCAGCTGGCGGGTAAAACAG	$(AGC)_7$	52	JOE	Blair et al. [25]
BM151	AF483867	*P. vulgaris* genomic clone D49	B08	CACAACAAGAAAGACCTCCT	TTATGTATTAGACCACATTACTTCC	$(TC)_{14}$	50	TAMRA	Gaitan-Solis et al. [52]

Table S4. Descriptive statistic of seed quantitative variables detected on 66 landraces from Lazio region. SL = seed length; SH = seed height; 100W = 100-seed weight; L/H ratio between seed length and height; SD= Standard Deviation, CV= Coefficient of Variation expressed as a percentage.

Variable	Mean	SD	CV %	Min		Max	
				value	landrace	value	landrace
SL	12.82	2.48	19.39	7.18	Tondino_VT	18.88	Cinelli_FR
SH	7.92	1.21	15.31	5.51	Tondino_VT	10.71	Zia Bettina_RI
100W	51.57	19.09	37.02	20.00	Tondino_VT	105.00	Zia Bettina_RI
L/H	1.63	0.29	17.97	1.15	Tondino_VT	2.37	Al burro_LT

Table S5. Genetic diversity parameters from the 12 SSR loci used for the analysis of the *P. vulgaris* collection from Lazio Region.

Locus	N_a[a]	N_e[b]	MAF[c]	H_o[d]	H_e[e]	F[f]	PIC[g]
X04001	3.0	1.754	0.697	0.000	0.430	1.000	0.348
X74919	2.0	1.677	0.719	0.013	0.404	0.967	0.322
X80051	13.0	4.114	0.459	0.037	0.757	0.951	0.743
AF483876	14.0	8.082	0.219	0.037	0.876	0.958	0.864
X60000	3.0	1.680	0.724	0.000	0.405	1.000	0.330
AF483902	7.0	3.783	0.313	0.031	0.736	0.958	0.689
SHP1B	4.0	3.698	0.342	0.018	0.730	0.976	0.680
AF483884	4.0	2.935	0.504	0.000	0.659	1.000	0.611
X61293	14.0	8.296	0.223	0.026	0.879	0.970	0.868
M75856	4.0	2.817	0.477	0.011	0.645	0.983	0.580
AZ044945	2.0	1.452	0.807	0.000	0.311	1.000	0.263
AF483867	5.0	2.736	0.482	0.000	0.635	1.000	0.570
**Mean**	6.3	3.585	0.497	0.014	0.622	0.977	0.572
**Total**	75.0						

[a]Number of observed alleles per locus; [b]effective number of alleles; [c]major allele frequency; [d]observed heterozygosity; [e]expected heterozygosity; [f]Inbreeding coefficient; [g]polymorphic information content.

Table S6. Genetic diversity parameters for the 66 landraces.

LANDRACE	N[a]	Nacc[b]	Na[c]	Ne[d]	Npa[e]	Ho[f]	He[g]
1 Cappellette_RM	4	1	1.000	1.000	1	0.000	0.000
2 Suricchio_2_FR	8	2	1.000	1.000	1	0.000	0.000
3 Pallino_RM	4	1	1.000	1.000	0	0.000	0.000
4 Regina Borl.Nano_RM	4	1	1.000	1.000	0	0.000	0.000
5 Pallinello giallo_LT	4	1	1.000	1.000	0	0.000	0.000
6 Al Burro_LT	4	1	1.000	1.000	0	0.000	0.000
7 Giallo_Nazzareno_RI	4	1	1.000	1.000	0	0.000	0.000
8 Borlotto_concetta_RI	4	1	1.000	1.000	0	0.000	0.000
9 Chiarinelli_RI	4	1	1.000	1.000	0	0.000	0.000
10 Corvaro_RI	4	1	1.000	1.000	0	0.000	0.000
11 Bianco Rieti_RI	4	1	1.000	1.000	0	0.000	0.000
12 Quaglia_RI	4	1	1.000	1.000	0	0.000	0.000
13 Borbone Leonessa_RI	4	1	1.000	1.000	0	0.000	0.000
14 Casette_RI	4	1	1.000	1.000	0	0.000	0.000
15 Regina_2_RI	4	1	1.000	1.000	0	0.000	0.000
16 Monichelle_2_RI	4	1	1.000	1.000	0	0.000	0.000
17 Pallottili_RI	4	1	1.000	1.000	0	0.000	0.000
18 Bottonc bianco_FR	8	2	1.000	1.000	0	0.000	0.000
19 Borlotto frasca_FR	4	1	1.000	1.000	0	0.000	0.000
20 Bottonc. Terelle_FR	4	1	1.000	1.000	0	0.000	0.000
21 Cann. Mosca_FR	4	1	1.000	1.000	0	0.000	0.000
22 Cinelli_FR	4	1	1.000	1.000	0	0.000	0.000
23 S.Pietro_FR	4	1	1.000	1.000	1	0.000	0.000
24 Nero_FR	4	1	1.000	1.000	0	0.000	0.000
25 Giallo stoppa_VT	4	1	1.000	1.000	0	0.000	0.000
26 Giallo quarant_VT	4	1	1.000	1.000	0	0.000	0.000
27 Giallo canarino_VT	4	1	1.000	1.000	0	0.000	0.000
28 Giallo_1_VT	4	1	1.000	1.000	0	0.000	0.000
29 Occhietto_VT	4	1	1.000	1.000	0	0.000	0.000
30 S.Anna_VT	4	1	1.000	1.000	0	0.000	0.000
31 Cerino_VT	4	1	1.000	1.000	0	0.000	0.000
32 Borlotto Montef_VT	4	1	1.000	1.000	0	0.000	0.000
33 Solfarino_VT	4	1	1.000	1.000	0	0.000	0.000
34 Tondino_VT	4	1	1.000	1.000	0	0.000	0.000
35 Bottone striato_FR	4	1	1.083	1.050	0	0.000	0.031
36 Cera_VT	8	2	1.083	1.083	0	0.000	0.042
37 Suricchio_1_RM	4	1	1.083	1.023	0	0.021	0.018
38 Regina_RM	4	1	1.083	1.023	0	0.021	0.018
39 Regina_LT	4	1	1.083	1.023	0	0.021	0.018
40 Zia Bettina_RI	4	1	1.083	1.023	0	0.021	0.018
41 Regina_3_RI	4	1	1.083	1.023	0	0.021	0.018
42 Cann. Atina_FR	24	6	1.083	1.083	0	0.010	0.042
43 Regina borlotto_LT	4	1	1.083	1.074	0	0.021	0.039
44 Mughetto_RI	4	1	1.083	1.074	1	0.021	0.039
45 Regina_1_RI	4	1	1.083	1.074	0	0.021	0.039
46 Monichelle_1_RI	4	1	1.083	1.074	0	0.021	0.039
47 Giallo_2_VT	4	1	1.083	1.074	0	0.021	0.039
48 Can.bian Pium_FR	16	4	1.167	1.022	0	0.010	0.019
49 Zeccuni_RI	8	1	1.250	1.204	0	0.010	0.111
50 Regina Nano Mar_RM	4	1	1.167	1.147	1	0.042	0.078
51 Carne_VT	4	1	1.167	1.147	0	0.042	0.078
52 S.Lorenzo_VT	4	1	1.250	1.230	0	0.042	0.120
53 Regina M.Equo_RM	4	1	1.250	1.120	0	0.063	0.076
54 Pelone_RI	16	4	1.167	1.060	0	0.010	0.043
55 Arsolana_RM	24	6	1.167	1.093	2	0.010	0.051
56 Romanesco_RM	8	2	1.250	1.118	0	0.031	0.070
57 Purgatorio_VT	20	5	1.333	1.219	3	0.008	0.112
58 Ciavat picc_VT	8	1	1.167	1.162	0	0.021	0.082
59 Cioncone_RM	12	3	1.250	1.132	0	0.028	0.086
60 Can.grig Pium_FR	16	4	1.333	1.276	1	0.021	0.130
61 Gentile_RI	12	3	1.417	1.195	1	0.028	0.129
62 Pisello_RI	16	4	1.333	1.160	1	0.031	0.107
63 Verdolino_VT	16	4	1.917	1.595	1	0.026	0.251
64 Can.S.Oliva_FR	16	4	1.667	1.338	0	0.031	0.191
65 Borbontino_RI	16	4	1.333	1.178	0	0.036	0.111
66 Can.ros Pium_FR	16	4	1.583	1.352	0	0.031	0.169

[a]Number of individual analyzed for each landrace; [b]Number of accessions for each landrace; [c]Number of alleles per locus; [d]Number of effective alleles; [e]Number of private alleles; [f]observed heterozygosity; [g]expected heterozygosity.

Table S7. Rare and private alleles detected on accessions of different landraces in homozygous (Hom) or heterozygous (Het) status.

LOCUS	Allele[a]	Frequency	Accession name[b]	Arsial code	Landrace	Status
**X04001**	165*	0.0132	Purgatorio_1 (4)	VE183_VT	Purgatorio	Hom
			Purgatorio_3 (2)	VE187_VT	Purgatorio	Hom
**X80051**	200	0.0285	Bott. Striato (4)	VE230_FR	Bott. Striato	Hom
			Nero (4)	VE238_FR	Nero	Hom
			Cann. Rosso Piumarola_3 (2)	VE120_FR	Cann. Rosso Piumarola	Hom/Het
			Cann. Rosso Piumarola_4 (3)	VE233_FR	Cann. Rosso Piumarola	Hom/Het
	204	0.0241	Chiarinelli (4)	VE263_RI	Chiarinelli	Hom
			Corvaro (4)	VE273_RI	Corvaro	Hom
			Zeccuni_2 (3)	3pg_RI	Zeccuni	Hom
	206	0.0285	Bott. Terelle (4)	VE287_FR	Bottoncino Terelle	Hom
			Purgatorio_1 (4)	VE183_VT	Purgatorio	Hom
			Purgatorio_3 (1)	VE187_VT	Purgatorio	Het
			S. Lorenzo (2)	VE425_VT	San Lorenzo	Hom
			Cioncone_3 (2)	VE243_RM	Cioncone	Hom
	214*	0.0339	Purgatorio_2 (4)	VE185_VT	Purgatorio	Hom
			Purgatorio_3 (4)	VE187_VT	Purgatorio	Hom/Het
			Purgatorio_4 (4)	VE422_VT	Purgatorio	Hom
			Purgatorio_5 (4)	VE184_VT	Purgatorio	Hom
	218	0.0351	Monichelle_1 (4)	6pg_RI	Monichelle_1	Hom
			Monichelle_2 (4)	8pg_RI	Monichelle_2	Hom
			Cinelli (4)	VE383_FR	Cinelli	Hom
			S. Pietro (4)	VE384_FR	San Pietro	Hom
	220	0.0329	Arsolana_2 (4)	VE469_RM	Arsolana	Hom/Het
			Arsolana_3 (4)	VE470_RM	Arsolana	Hom
			Arsolana_4 (4)	VE471_RM	Arsolana	Hom/Het
			Pallino (4)	VE215_RM	Pallino	Hom
	224	0.0153	Cappellette (4)	VE213_RM	Cappellette	Hom
			Mughetto (3)	VE269_RI	Mughetto	Hom/Het
			Romanesco_1 (1)	VE217_RM	Romanesco	Het
	228	0.0285	Arsolana_1 (4)	VE468_RM	Arsolana	Hom
			Arsolana_5 (4)	VE478_RM	Arsolana	Hom
			Arsolana_6 (4)	VE244_RM	Arsolana	Hom
			Mughetto (1)	VE269_RI	Mughetto	Hom

LOCUS	Allele[a]	Frequency	Accession name[b]	Arsial code	Landrace	Status
**AF483876**	186	0.0175	Solfarino (4)	VE196_VT	Solfarino	Hom
			Ciavattone piccolo_1 (3)	VE179_VT	Ciavattone piccolo	Hom/Het
			Ciavattone piccolo_2 (1)	VE180_VT	Ciavattone piccolo	Hom
	224	0.0417	Pisello_1 (4)	VE294_RI	Pisello	Hom
			Pisello_2 (3)	VE295_RI	Pisello	Hom/Het
			Pisello_3 (2)	VE385_RI	Pisello	Hom/Het
			Pisello_4 (4)	VE386_RI	Pisello	Hom
			Gentile_1 (3)	VE274_RI	Gentile	Hom/Het
			Cann. S.Oliva_1 (3)	VE132_FR	Cannellino S. Oliva	Hom/Het
			Cann. S.Oliva_2 (1)	VE135_FR	Cannellino S. Oliva	Hom
			Cann. S.Oliva_4 (1)	VE137_FR	Cannellino S. Oliva	Hom
	228*	0.0175	Suricchio_2 (4)	VE458_FR	Suricchio_2_FR	Hom
			Suricchio_3 (4)	VE459_FR	Suricchio_2_FR	Hom
	238	0.0351	Romanesco_1 (4)	VE217_RM	Romanesco	Hom
			Romanesco_2 (4)	VE222_RM	Romanesco	Hom
			Regina Borlotto Nano_RM (4)	VE225_RM	Regina Borlotto Nano_RM	Hom
			Cinelli (4)	VE383_FR	Cinelli	Hom
	242	0.0175	Regina_LT (4)	VE483_LT	Regina_LT	Hom
			Regina Borlotto_LT (4)	VE487_LT	Regina Borlotto_LT	Hom
	250*	0.0087	Reg Nano Marano Equo (4)	VE226_RM	Regina Nano Marano Equo	Hom
	256*	0.0131	Cann. Grigio Pium_1 (1)	VE128_FR	Cannellino Grigio Piumarola	Hom
			Cann. Grigio Pium_2 (4)	VE129_FR	Cannellino Grigio Piumarola	Hom
			Cann. Grigio Pium_4 (1)	VE232_FR	Cannellino Grigio Piumarola	Hom
**X60000**	141*	0.0087	San Pietro (4)	VE384_FR	San Pietro	Hom
**AF483902**	164*	0.0219	Gentile_1 (4)	VE274_RI	Gentile	Hom
			Gentile_2 (4)	VE423_RI	Gentile	Hom
			Gentile Labro (2)	13pg_RI	Gentile	Hom
	170	0.0417	Pisello_1 (2)	VE294_RI	Pisello	Hom/Het
			Pisello_2 (3)	VE295_RI	Pisello	Hom
			Pisello_3 (4)	VE385_RI	Pisello	Hom
			Pisello_4 (4)	VE386_RI	Pisello	Hom
			Bianco di Rieti (4)	VE275_RI	Bianco di Rieti	Hom
			Giallo_2_VT (3)	VE248_VT	Giallo_2_VT	Hom/Het
	180*	0.0022	Verdolino_3 (1)	VE191_VT	Verdolino	Hom

LOCUS	Allele[a]	Frequency	Accession name[b]	Arsial code	Landrace	Status
**AF483902**	186*	0.0526	Arsolana_1 (4)	VE468_RM	Arsolana	Hom
			Arsolana_2 (4)	VE469_RM	Arsolana	Hom
			Arsolana_3 (4)	VE470_RM	Arsolana	Hom
			Arsolana_4 (4)	VE471_RM	Arsolana	Hom
			Arsolana_5 (4)	VE478_RM	Arsolana	Hom
			Arsolana_6 (4)	VE244_RM	Arsolana	Hom
**X61293**	160*	0.0109	Purgatorio_2 (1)	VE185_VT	Purgatorio	Het
			Purgatorio_3 (4)	VE422_VT	Purgatorio	Hom
	168	0.0417	Romanesco_1 (4)	VE217_RM	Romanesco	Hom
			Romanesco_2 (4)	VE222_RM	Romanesco	Hom
			Regina_2_RI (4)	5pg_RI	Regina_2_RI	Hom
			Cerino (4)	VE340_VT	Cerino	Hom
			Cann. Rosso Piumarola_2 (3)	VE117_FR	Cannellino Rosso Piumarola	Hom
	178	0.0406	Cann. Rosso Pimarola_4 (4)	VE233_FR	Cannellino Rosso Piumarola	Hom
			Cera_1 (4)	VE427_VT	Cera	Hom
			Regina_LT (4)	VE483_LT	Regina_LT	Hom
			S. Pietro (4)	VE384_FR	San Pietro	Hom
			Regina M. Equo (3)	VE224_RM	Regina Marano Equo_RM	Hom/Het
	182*	0.0087	Cappellette (4)	VE213_RM	Cappellette	Hom
	188*	0.0263	Pisello_1 (4)	VE294_RI	Pisello	Hom
			Pisello_2 (1)	VE295_RI	Pisello	Hom
			Pisello_3 (3)	VE385_RI	Pisello	Hom
			Pisello_4 (4)	VE386_RI	Pisello	Hom
	194*	0.0087	Mughetto (4)	VE269_RI	Mughetto	Hom
**M75856**	149	0.0263	Suricchio_1 (4)	VE457_RM	Suricchio_1_RM	Hom
			Regina_1_RM (4)	VE216_RM	Regina_RM	Hom
			Regina Marano Equo_RM (4)	VE224_RM	Regina Marano Equo_RM	Hom

LOCUS	Allele[a]	Frequency	Accession name[b]	Arsial code	Landrace	Status
**AF483867**	151	0.0438	Zia Bettina (4)	4pg_RI	Zia Bettina	Hom
			Monichelle_2 (4)	8pg_RI	Monichelle_2	Hom
			Giallo Canarino (4)	VE198_VT	Giallo Canarino	Hom
			Giallo_2 (4)	VE248_VT	Giallo_2	Hom
			S. Anna (4)	VE258_VT	S. Anna	Hom
	155*	0.0526	Arsolana_1 (4)	VE468_RM	Arsolana	Hom
			Arsolana_2 (4)	VE469_RM	Arsolana	Hom
			Arsolana_3 (4)	VE470_RM	Arsolana	Hom
			Arsolana_4 (4)	VE471_RM	Arsolana	Hom
			Arsolana_5 (4)	VE478_RM	Arsolana	Hom
			Arsolana_6 (4)	VE244_RM	Arsolana	Hom

[a] The asterisk indicates the private alleles

[b] In brackets are indicated the individuals for each accession with that particular allele

Table S8. Analysis of molecular variance among and within landraces.

Source	df	SS	Est. Var.	%	*Φ*-statistic	P(*Φ*)
Among landraces	65	3062.72	3.41	89	0.887	<0.001
Within landraces	846	341.91	0.40	11		
Total	911	3404.63	3.82	100		

df: degree of freedom; SS: sum of square calculated from a square genetic distance matrix; Est.Var.: estimated variance. P(*Φ*)–*Φ*-statistic probability level after 999 permutations.

Table S9. Number of alleles and different genotypes detected in the 32 landraces showing intra-genetic diversity.

Landrace	N. of accession analysed	Loci	N. of alleles/locus	Number of different genotypes
Arsolana	6	X80051	2	5
		AF483876	2	
Cioncone	3	X80051	2	8
		X61293	2	
		M75856	2	
Romanesco	2	X74919	2	5
		X80051	2	
		AF483884	2	
Suricchio1_RM	1	X74919	2	2
Regina_RM	1	SHP1B	2	2
ReginaME_RM	1	AF483902	2	4
		SHP1B	2	
		X61293	2	
ReginaNME_RM	1	SHP1B	2	4
		M75856	2	
Regina_LT	1	AF483902	2	2
Reginabor_LT	1	AF483902	2	3
Pelone	4	X74919	2	5
		AF483902	2	
Borbontino	4	X80051	2	12
		AF483902	2	
		X61293	2	
		M75856	2	
Pisello	4	AF483876	2	9
		AF483902	2	
		SHP1B	2	
		X61293	2	
Gentile	3	X74919	2	9
		AF483876	2	
		AF483902	2	
		SHP1B	2	
		AF483884	2	
Mughetto	1	X80051	2	3
Regina1_RI	1	AF483876	2	3
Zeccuni	2	X04001	2	4
		X80051	2	
		X61293	2	
Zia Bettina	1	AF483802	2	2

(**Table S9 continued**)

Landrace	N. of accession analysed	Loci	N. of alleles/locus	Number of different genotypes
Monichelle1_RI	1	SHP1B	2	3
Regina3_RI	1	AF483802	2	2
Cann.Atina	6	AF483876	2	3
Can.bianco Pium	4	AF483876	2	4
		SHP1B	2	
Can.grigio Pium	4	X80051	2	8
		AF483876	2	
		AF483884	3	
Can.S.Oliva	4	AF483876	3	10
		X60000	2	
		AF483902	2	
		SHP1B	2	
		AF483884	2	
		X61293	2	
Can.rosso Pium	4	X80051	2	13
		AF483876	3	
		AF483902	2	
		AF483884	2	
		X61293	3	
Bottone striato	1	AF483902	2	2
Purgatorio	5	X04001	2	6
		X80051	2	
		X61293	3	
Verdolino	4	X04001	2	10
		X80051	2	
		AF483876	3	
		AF483902	3	
		X61293	4	
		AF483867	2	
Giallo2_VT	1	AF483902	2	3
Ciavat picc	2	AF483876	2	6
		X61293	2	
S.Lorenzo	1	X80051	2	4
		AF483902	2	
		AF483884	2	
Cera	2	X61293	2	2
Carne	1	X80051	2	4
		X61293	2	
