# Supplementary figures and images for: Genetic Diversity and Population Structure of Common Bean (Phaseolus vulgaris L.) Landraces in the Lazio Region of Italy
Source: Plants (Basel). 2023 Feb 7;12(4):744. doi: 10.3390/plants12040744 (PMC9968208; doi:10.3390/plants12040744)

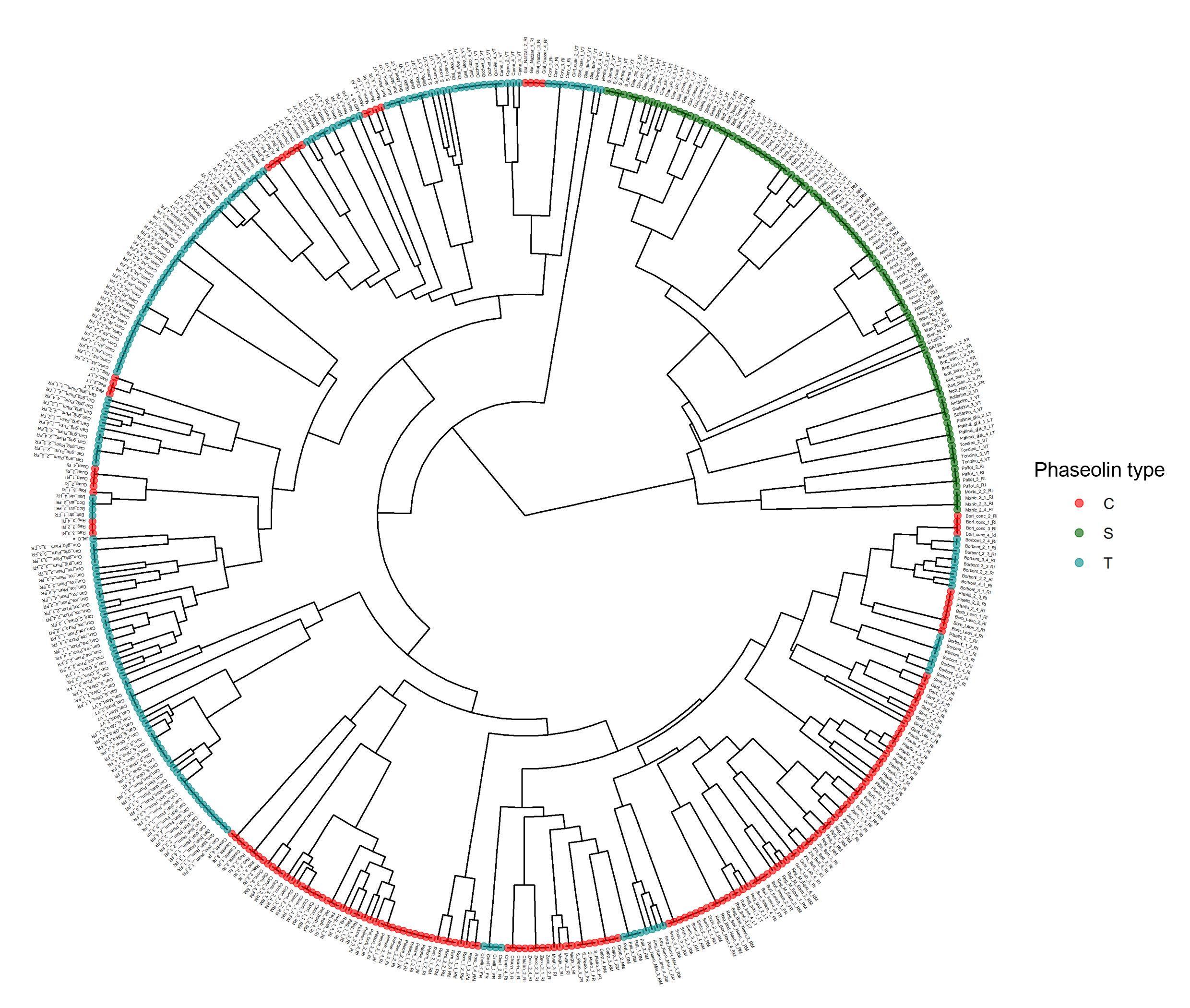

Supplement: Supplementary file 1 [file plants-12-00744-s001.zip › plants-2143655-supplementary/Figure S5.tif]
